# The Effects of Various Types of Physical Exercise on Health Outcomes in Older Adults with Depression: A Systematic Review and Meta-Analysis of Controlled Trials

**DOI:** 10.1155/2024/9363464

**Published:** 2024-06-19

**Authors:** Samaher Alowaydhah, Ishanka Weerasekara, Sarah Walmsley, Jodie Marquez

**Affiliations:** ^1^College of Health, Medicine and Wellbeing, The University of Newcastle, Newcastle, Australia; ^2^College of Applied Medical Science, Jouf University, Sakaka, Saudi Arabia; ^3^Faculty of Health and Social Sciences, Western Norway University of Applied Sciences 5063, Bergen, Norway; ^4^School of Allied Health Science and Practice, Faculty of Health and Medical Sciences, The University of Adelaide, Adelaide, SA 5005, Australia; ^5^Hunter Medical Research Institute, New Lambton, Australia

## Abstract

**Methods:**

An electronic search of five databases was used to retrieve controlled studies investigating health-related outcomes associated with any type of PE, in adults over 65 years with depression. Where possible, data were analyzed in meta-analyses or else reported narratively. *Results and Discussion*. Fifteen studies were included in the review. All 15 studies had data available for meta-analysis, yet heterogeneity in exercise types and outcome measures limited synthesis. When data for all types of exercise were pooled together, significant improvements were found in depression (SMD = 0.52; CI = 0.07, 0.97; *p* = 0.02), physical health and function (SMD = 0.44, CI = 0.14, 0.74; *p* = 0.004), anxiety (SMD = 0.23; CI = 0.04, 0.42; *p* = 0.02), and self-efficacy (SMD = 0.97; CI = 0.32, 1.61; *p* = 0.003). When data were pooled according to exercise type, positive effects were demonstrated for aerobic exercise on depression (SMD = 0.49; CI = −0.16, 0.8; *p* = 0.003), tai chi/qigong on depression (SMD = 0.89; CI = −0.14, 1.63; *p* = 0.02), and self-efficacy (SMD = 6.70; CI = −0.90, 12.50; *p* = 0.02) and multicomponent exercise demonstrated positive effects on physical function (SMD = 0.49; CI = 0.12, 0.87; *p* = 0.009) and the physical health component of quality of life (SMD = 0.88; CI = 0.10, 1.65; *p* = 0.03). No health-related benefits were revealed from the combined findings of the two studies investigating strengthening exercises.

**Conclusion:**

Lack of consistency regarding exercise types, dosage, and outcomes presents challenges in the evidence. In general, exercise is beneficial across a range of health-related variables. Multicomponent, aerobic, and tai chi forms of exercise appear most likely to reap benefits in depressed older adults; however, the type of benefit is determined by the type of exercise and should be considered when recommending a regime. This trial is registered with CRD42020173465.

## 1. Introduction

The number of older adults aged 65 years represents more than a third of the world population [1], with the global population growth in this age group increasing from 727 million in 2020 to 1.5 billion by 2050 [2]. This results in increased pressure on health and social services with older adult healthcare usage per capita estimated to be 3 to 5 times higher than that of young adults [3]. Statistics from Western countries indicate that up to 50% of the overall health budget is spent on providing care to older adults [4], with predictions for significant increases in residential care spending [5]. Therefore, strategies to maintain the independence and health of this group are crucial to combat the escalating costs that are borne by the individual and society.

The number of chronic diseases rises concurrently with age [6], with depression being the most prevalent mental disease affecting over 350 million older adults globally [7, 8]. The World Health Organization states that depression is the primary contributor to mortality and poor quality of life in older adults [8] and is correlated with various conditions such as cardiovascular disease [9], diabetes [10], and acute coronary syndrome [11]. Depression is a mood disorder that ranges from typical, fleeting periods of sadness experienced in everyday life to a clinical syndrome characterized by prolonged and intense symptoms that significantly deviate from normality [12].

Physical exercise (PE) has been demonstrated to have positive impacts on psychological domains especially depression in older adults [13, 14]. The National Institute for Health and Clinical Excellence recommends incorporating group exercise, particularly aerobic exercise, into the treatment of depression. They suggest engaging in organized and supervised moderate-intensity exercise routines, more than one session per week, for 10 weeks [15]. Similarly, the Royal Australian and New Zealand College of Psychiatrists have included regular exercise in their Clinical Practice Guidelines for mood disorders. They add that although the optimal amount and type of exercise are yet to be determined, regular aerobic and resistance exercises are likely optimal [16]. For older adults in general, the Physical Activity Guideline for Americans suggests at least 150 minutes of moderately intense exercise or a minimum of 75 minutes of vigorous exercise per week. For those with chronic conditions, they should be as physically active as their abilities and conditions allow [17].

In older adults with depression, research has shown that PE may have benefits in ameliorating not just the mood disorder, but other health indicators as well [18]. Five previous reviews have been conducted investigating the effect of physical exercise in older adults with depression. One previous systematic review investigated the effect of different types of exercise programs on depression and concluded that mind-body exercise had the greatest impact on reducing depressive symptoms. This review is limited in that it restricted its scope to only three types of exercise and excluded multicomponent exercise programs. The authors justify that this was to avoid any potential synergistic effect on depressive symptoms, within a single intervention group [19]. However, the majority of community and clinically run programs for older adults offer multicomponent exercise; therefore, inclusion of this type of program is warranted. A further three reviews measured the effect of exercise on one outcome in isolation, depression, limiting the ability to draw holistic conclusions about the value of exercise in this population [20–22]. The final review was limited in that it restricted both exercise types and outcomes of interest to a narrow focus [23].

These limitations produce a lack of coherence in the literature. This systematic review is aimed at collating and synthesizing evidence regarding the prescription of exercise in older adults with depression to determine the effectiveness of specific types of exercise on a spectrum of health outcomes. The objective was twofold: initially, to ascertain the advantages of engaging in PE concerning specific health indices such as (1) physical health and functioning, (2) cognitive function, and (3) mental health and overall wellbeing among older adults with depression and, secondly, to identify the specific forms of exercise that offer the greatest benefits to this particular demographic.

## 2. Method

This review is one component of a broader review aimed at developing exercise recommendations for older adults, and the protocol for this systematic review was registered with the International Prospective Register of Systematic Reviews on 28 April 2020 (CRD42020173465). The overarching review encompasses specific subgroups of individuals who have common medical conditions in older populations, such as cognitive impairment, orthopedic conditions, and neurological diseases. The findings related to these subgroups will be presented in separate reports. This review focuses on older adults who were recruited into exercise studies with the fundamental criteria of being depressed. This review adhered to the guidelines outlined in the Preferred Reporting Items for Systematic Reviews and Meta-Analyses (PRISMA) statement [24].

### 2.1. Information Sources

The search strategy was developed using key terms related to “exercise” and “geriatric”. Medline, Cochrane, Embase, CINAHL, and Scopus databases were comprehensively searched. The study did not use a strict methodological filter to be inclusive, but it set a time limit for studies published after 1989 to match current exercise practices. Research during the COVID pandemic was not included because programs were often modified, making them inconsistent for meta-analysis in terms of recruitment, implementation, adherence, and outcomes. The search strategy used for MEDLINE is provided as an example in supplementary material (available here).

### 2.2. Eligibility Criteria

The inclusion criteria for selected articles were (a) articles written in English and limited to human adults (≥65 years) on average, (b) study design limited to randomized controlled studies, and (c) physical exercise intervention compared with control. Exclusion criteria included (a) those published before 1990; (b) those published in languages other than English; (c) those focusing on cardiac, respiratory, and postsurgery populations; and (d) those with study designs such as protocols, systematic reviews, editorials, commentaries, reports, conference abstracts, case studies, and case series.

The PICO format, consisting of four stages (population, intervention, comparison, and outcome), was used for this analysis. The following section provides detailed explanations of each aspect.

### 2.3. Population

The study primarily concentrated on individuals of either gender who were aged 65 or above. These individuals were classified as depressed older adults, based on the original authors' definitions. In cases where the study population included individuals of various age groups, studies were included if the average age of the population was greater than 65 years. Studies that focused on populations with cardiac or respiratory diseases, recent fractures, or individuals who were in the postsurgery phase were excluded from the review since these subgroups require specific exercise considerations.

### 2.4. Intervention

We included studies examining any form of physical exercise intervention as described by the definition of a planned, organized, and repeated physical activity that seeks to enhance or sustain one or more physical fitness components [25]. Both individual exercise and group settings were considered in this analysis. However, studies that involved specific programs targeted at cardiac/respiratory interventions or fall prevention were excluded from the review.

### 2.5. Comparison

Only studies that included a control condition were included in the analysis. The control condition could consist of usual care (such as occupational therapy or no care), different types of interventions (such as cognitive behavioral therapy), or wait-list control. Studies that compared different types of exercise interventions without a nonexercise control condition and compared exercise intervention with depressed medication were excluded from the review.

### 2.6. Outcome Measures

Studies focusing on health-related outcomes were considered for inclusion. This encompassed quality of life measured by validated tools such as Older People Quality of Life Questionnaire (OPQoL-brief) [26], cognitive function such as the Mini-Mental State Examination (MMSE) [27], depression (e.g., Geriatric Depression Scale (GDS) [28], mood such as Hospital Anxiety and Depression Scale (HADS) [29], and pain intensity such as Visual Analog Scale) [30], and functional outcomes such as activity daily living (ADL). Studies which did not include any one of these outcomes were excluded. In assessing the quality of life, we included subscores of physical health and mental health in the instances where studies did not report the composite total for analysis. If a study incorporated multiple assessments of a similar outcome, such as both the Geriatric Depression Scale (GDS) and Hospital Anxiety and Depression Scale (HADS) to evaluate mood, we used the data from the tool reported to be the primary outcome measure. In the case that this was not stated, the data from the tool that appeared more frequently across the included studies was used in the analysis. To control for potential bias, sensitivity analyses were conducted.

### 2.7. Selection Process

Following the removal of duplicate studies, two reviewers (SA, IW) independently evaluated the titles and abstracts and subsequently the full texts, to determine eligibility. Any disagreements between the reviewers were resolved through discussion with a third reviewer (JM). The screening process was facilitated by Covidence (a systematic review software, Veritas Health Innovation, Melbourne, Australia).

### 2.8. Data Collection Process

Data were extracted to a spreadsheet, and all relevant details including study title, authors, publication year, published country, sample size, sex distribution, study design, participants, intervention, comparison, and outcomes and their significance were recorded. Data extraction was done by one author (SA) and cross-checked by a third author. The methodological quality of individual studies was determined using the PEDro scale. This scale is widely accepted as a reliable [31] and valid [32] tool for evaluating the methodological quality of clinical trials, particularly in the context of physiotherapy and rehabilitation research.

### 2.9. Synthesis Methods

A descriptive analysis was conducted on the extracted data, and the findings were presented in tables and graphs to address the research question. Whenever possible, comparable study data in terms of participant characteristics, intervention, and outcome measures were pooled, and meta-analyses were performed using RevMan software. The appropriate model (fixed or random effect) was selected based on the level of heterogeneity, which was assessed using the *I*^2^ statistic whereby higher scores represent higher levels of heterogeneity [33]. If the *I*^2^ value exceeded 30%, a random-effects model was applied to account for the intertrial heterogeneity. In the instance where different tools were used to measure the same outcome, the standard mean difference was calculated. This review followed the standard interpretation of effect sizes as small (0.2), medium (0.5), and large (0.8) [33]. In the instances where more than one tool was used to assess the outcome, we conducted sensitivity analyses by substituting data from one measurement tool for the others reported in the study to test the strength of the findings.

## 3. Results

### 3.1. Study Selection

**Figure 1:**
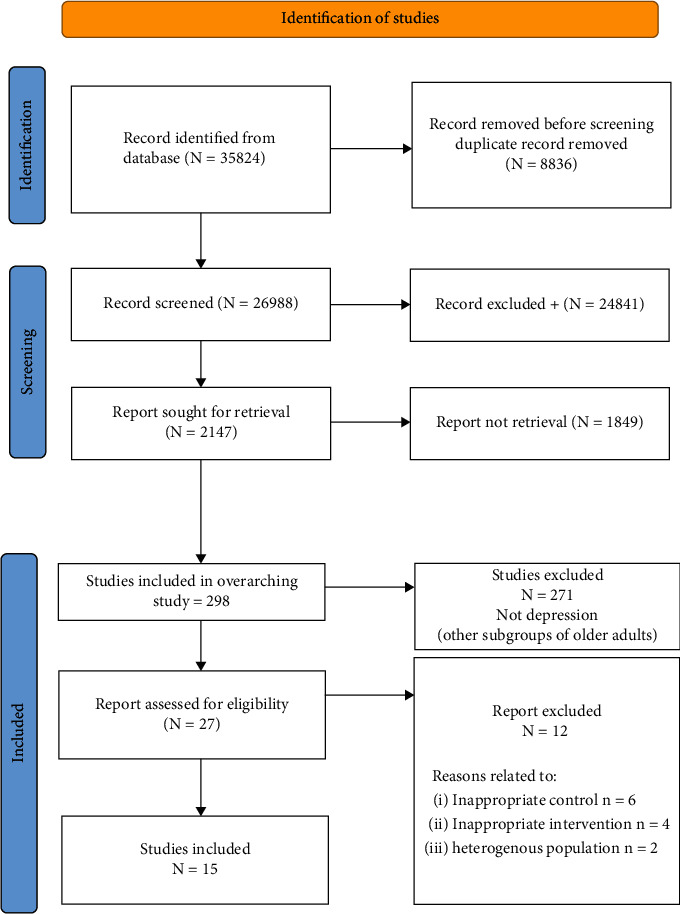
Flow of studies through the review.

The online search identified 35824 relevant citations, of which 8836 were duplicates. After their removal, 26988 articles were screened based on title and abstract. Altogether, 2147 studies were included in the full-text screen, of which 1849 articles were excluded. This left 298 articles related to exercise in older adults; in total, 15 studies were relevant for the subgroup of older adults with depression reported in the review. Please see Figure 1 for the flow of the review process.

### 3.2. Quality of the Included Studies

The 15 studies that met all the inclusion criteria demonstrated a range of methodological quality, from low to high (range 3-8), with an average PEDro score of 5.6 out of 10. Most studies (86.6%) used randomization, but only 26.6% reported concealed allocation. Among the quality assessment criteria, blinding received the poorest scores. No studies reported participant blinding or blinding of the therapists. For a detailed overview of the quality assessment scores, please refer to Table 1.

### 3.3. Study Characteristics

Fifteen studies involving 2100 older adults with depression were included in the review. The average age of participants was 76.7 years. Participants were recruited from a range of sources. Nine of these studies sourced participants from community settings [34–42], three from residential care facilities [43–45], and three from medical facilities [46–48].

The studies were conducted throughout the world with the highest frequency in Asia [36, 37, 40, 42, 44, 48]; four in the United States [34, 35, 39, 41]; and one study each in the UK [45], Italy [46], Australia [47], New Zealand [38], and Turkey [43].

The majority of studies provided multicomponent exercise interventions which included strength, flexibility/stretching, balance, coordination, and aerobic exercises [34–38, 40, 43, 45]. Strengthening exercises were applied in two studies [41, 47]. Similarly, qigong exercise was reported in two studies [44, 48]. Aerobic exercise [42, 46] and tai chi [39] were each investigated in single studies. The duration of the treatment and the total dose of exercise varied greatly between studies. The interventions lasted a minimum of 10 weeks [39, 43, 47], to a maximum of 52 weeks [45]. Individual session duration ranged from 30 minutes [44] to 120 minutes [39].  Table 2 provides a full description of the included studies; Table 3 describes the results of each study type and their outcomes.

### 3.4. Effect of Exercise (of Any Type) on Older Adults with Depression

**Figure 2:**
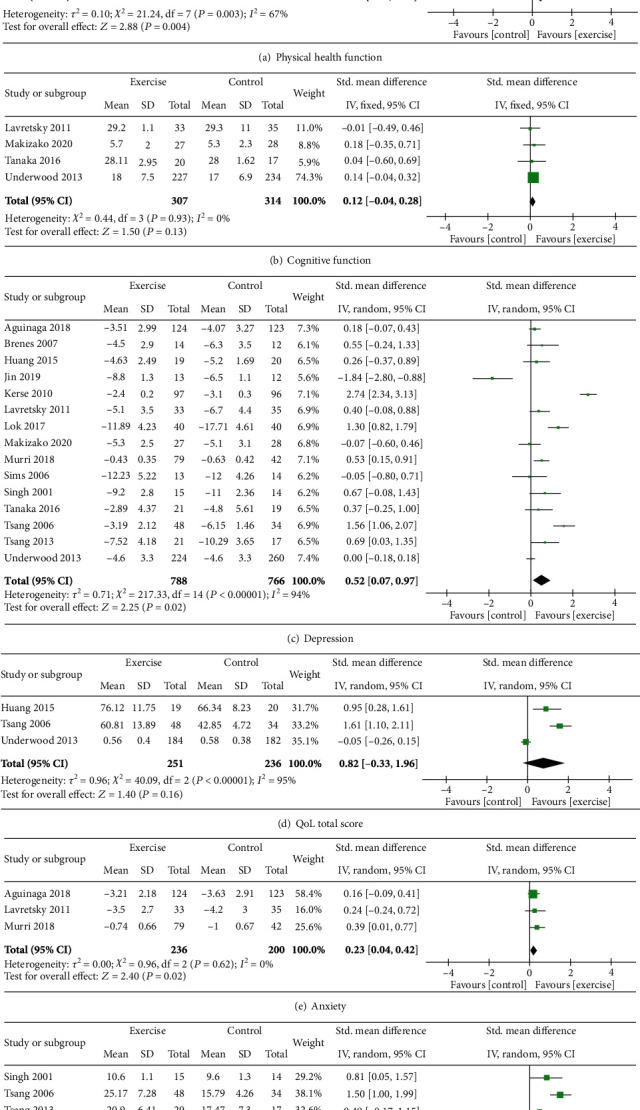
Effect of physical exercise (of any type) on older adults with depression.

All 15 studies investigated the effects of exercise on depression. When the data were pooled from 1554 participants, significant improvements were observed in physical health and function (SMD = 0.44; CI = 0.14, 0.74; *p* = 0.004). Similarly, positive effects of exercise were demonstrated in the outcomes of depression (SMD = 0.52; CI = 0.07, 0.97; *p* = 0.02), anxiety (SMD = 0.23; CI = 0.04, 0.42; *p* = 0.02), and self-efficacy (SMD = 0.97; CI = 0.32, 1.61; *p* = 0.003). No significant benefits were found for cognitive function (SMD = 0.12; CI = −0.04, 0.28; *p* = 0.13) or for quality of life (SMD = 0.82; CI = −0.33, 1.96; *p* = 0.16). Please refer to Figure 2.

#### 3.4.1. Effect of Multicomponent Exercise

Programs incorporating different components of exercise training were the most common form of exercise program that was investigated. This constituted 8 of the totals of 15 included studies.

**Figure 3:**
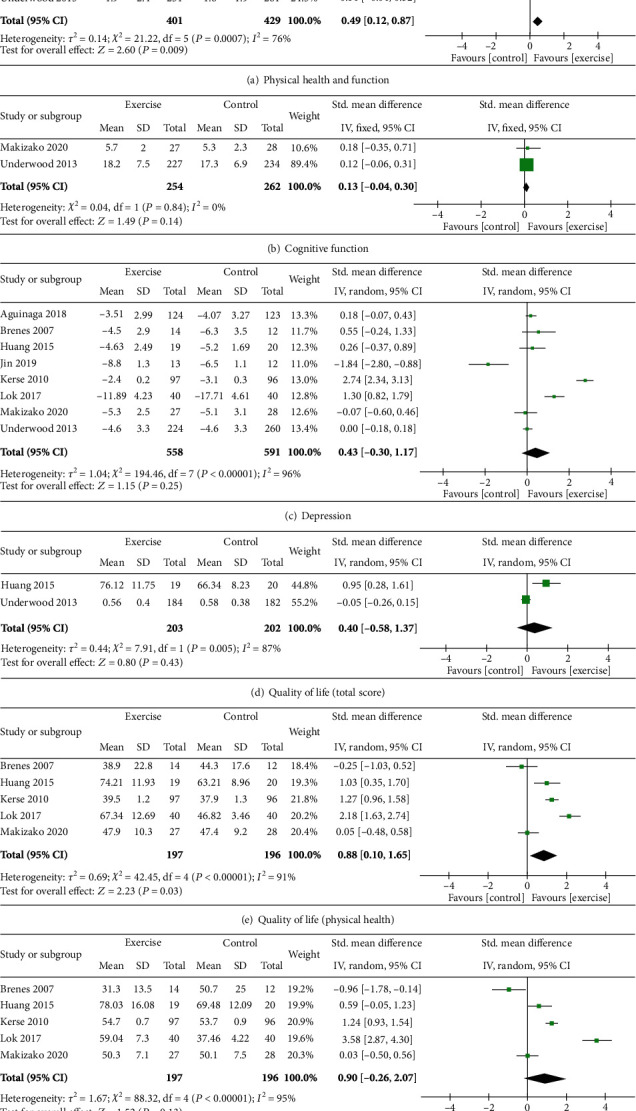
Effect of multicomponent exercise on older adults with depression.

*(1) Physical Health and Function*. Physical health was investigated in 7 studies, and improvement was reported in 5 of these studies [34–37, 40]. Data was available for meta-analysis for 6 studies using 6MWT, SPPS, and 2MWT [35–38, 40, 45] with a total of 830 participants. A significant benefit of exercise for physical health was demonstrated (SMD = 0.49; CI = 0.12, 0.87; *p* = 0.009). See Figure 3(a). In this meta-analysis, four studies used additional measures to assess physical health. Four of the studies included in the meta-analysis used additional tools to assess physical health [35, 37, 38, 40]. When sensitivity analyses were conducted by substituting these outcomes, the significance of the finding remained (*p* < 0.05).

*(2) Cognitive Function*. Two [40, 45] studies investigated the effects of multicomponent exercise on cognitive function using word list memory (delay) and MMSE. When the data were pooled from these studies of 516 participants, no significant effect was found (SMD = 0.13; CI = −0.04, 0.30; *p* = 0.14). See Figure 3(b).

*(3) Mental Health and Wellbeing*. Eight studies investigated the effect of multicomponent exercise on depression, and data were available from all studies for meta-analysis [34–38, 40, 43, 45]. Pooled data from 1149 participants from different depression tools, such as HADS, GDS, and BDI, revealed no positive effect of multicomponent exercise on mental health (SMD = 0.43; CI = −0.30, 1.17; *p* = 0.25). See Figure 3(c).

*(4) Quality of Life*. Six of the included studies investigated the effect of multicomponent exercise on quality of life, and 3 of these reported benefits [35, 36, 43]. Data were available for meta-analysis from 2 studies [36, 45] with 405 participants to show no significant benefit (SMD = 0.40; CI = −0.58, 1.37; *p* = 0.43). See Figure 3(d).

In some studies, quality of life was measured with the 36-Item Short Form Survey (SF-36), and component scores for physical and mental health were reported. A significant benefit of multicomponent exercises on the physical health component of quality of life was demonstrated when data were pooled for 393 participants [35, 36, 38, 40, 43] (SMD = 0.88; CI = 0.10, 1.65; *p* = 0.03). See Figure 3(e). Conversely, no positive benefit was demonstrated in the mental health component (SMD = 0.90; CI = −0.26, 2.07; *p* = 0.13). See Figure 3(f).

*(5) Anxiety*. One study explored the effect of multicomponent exercise on anxiety. The authors report that both the exercise and control groups had reduced anxiety at the conclusion of the study, but no significant between-group differences were evident in the Hospital Anxiety and Depression Scale (HADS) (intervention mean change = 2.90, control mean change = 0.73, and *p* = 0.28) [34].

#### 3.4.2. Effect of Aerobic Exercise

Two studies investigated the effects of aerobic type exercise on health-related outcomes in older adults with depression.

*(1) Physical Health and Function*. One study [42] investigated the effect of 3 months of Japanese drumming exercise, once a week for 40 minutes on physical function to reveal a positive effect on the shuttle stamina test by walking (mean change for intervention from baseline = 18.6; control group = −3.6, *p* = 0.002) and hand grip strength (mean change for intervention = −0.5; control group = −5.0, *p* = 0.001).

*(2) Cognitive Function*. This same study [42] assessed the effects of aerobic exercise on cognition by using the Mini-Mental State Examination (MMSE). No significant benefit of exercise, compared to usual activity, was revealed (median *n* change for intervention from baseline = 1.5 and control = 1, *p* = 0.332).

**Figure 4:**
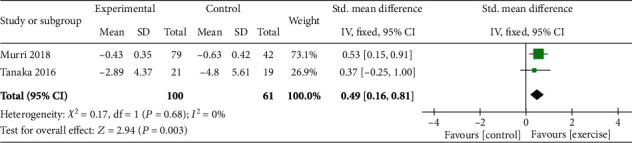
Effect of aerobic exercise on older adults with depression.

*(3) Mental Health and Wellbeing*. Both studies with a combined total of 161 participants [42, 46] assessed the merits of aerobic exercise on depression. Meta-analysis revealed a significant benefit compared to control (SMD = 0.49; CI = −0.16, 0.81; *p* = 0.003). See Figure 4.

*(4) Anxiety*. One study evaluated the effect of aerobic exercise on anxiety. No significant effect on this outcome was reported as measured on the Hamilton Depression Rating Scale (HAM-D) (mean change from baseline, for the intervention group = 0.99; control = 0.75, *p* = 0.06) [46].

#### 3.4.3. Effect of Strengthening Exercise

Two studies investigated the effect of strengthening exercise on health-related outcomes in older adults with depression.

*(1) Physical Health and Function*. One study assessed the effects of strengthening exercises on physical health by using the Human Activity Profile (HAP MAS). No significant benefit was observed (mean change for the intervention group from baseline = 2.57; control = 1.7, *p* = 0.07) [47].

**Figure 5:**
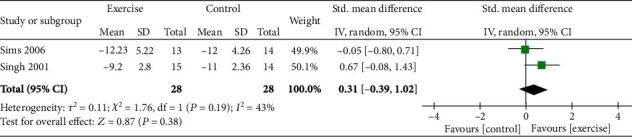
Effect of strengthening exercise on older adults with depression.

*(2) Mental Health and Wellbeing*. Two studies assessed the effect of strength training on depression using the Geriatric Depression Scale (GDS) and Beck Depression Inventory (BDI) tools [41, 47]. When the data for 56 participants were pooled for meta-analysis, no difference with control was found (SMD = 0.31; CI = −0.39, 1.02; *p* = 0.38). See Figure 5.

*(3) Quality of Life*. Quality of life was assessed using the World Health Organization Quality of Life (WHOQoL) tool. In one study, there was no significant effect in the domain of physical (mean change for intervention group from baseline = 0.41; control = 1.09, *p* = 0.36) or psychological (mean change for intervention group from baseline = 0.52; control = 0.18, *p* = 0.66) quality of life [47].

#### 3.4.4. Effect of Tai Chi/Qigong Exercises

Three studies assessed the effect of tai chi or qigong exercise on depressed older adults.

*(1) Physical Health and Function*. Of the three studies that evaluated the effects of tai chi/qigong exercises [39, 44, 48], only one assessed the outcome of physical health and functioning (hand grip strength) [48]. A statistically significant difference was reported between the exercise and control groups (the mean difference of right-hand strength for the intervention group from baseline and postintervention = 1.7 with an average increase and for control = 1.5 with an average decrease, *p* = 0.03). The data was not compatible with the meta-analysis.

*(2) Cognitive Function*. A single study assessed the effect of 10 weeks of tai chi for 120 minutes once a week to reveal a nonpositive benefit on cognitive function (MMSE) (mean change for the intervention group from baseline = 0; control = 0.3, *p* = 0.24) [39].

**Figure 6:**
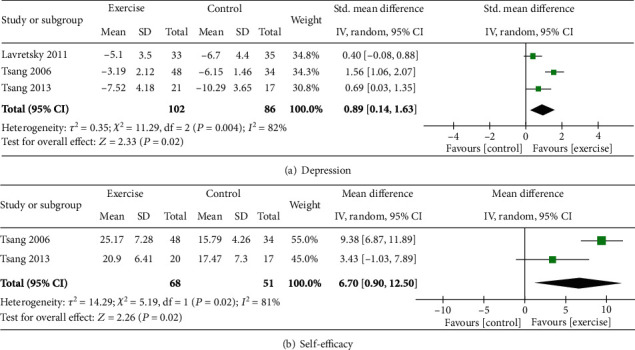
Effect of tai chi/qigong exercise on older adults with depression.

*(3) Mental Health and Wellbeing*. Data for 188 participants were pooled together, to demonstrate the positive benefit of tai chi and qigong exercise in improving mental health (SMD = 0.89; CI = −0.14, 1.63; *p* = 0.02) [39, 44, 48]. See Figure 6(a).

*(4) Quality of Life*. Two studies assessed the benefit of tai chi/qigong exercise on quality of life. One study reported improvement as measured by the 36-Item Short Form Health Survey (SF-36) for physical function (mean change for intervention group from baseline = 3.7; control = 0.3, *p* = 0.02), emotional function (mean change for the intervention group from baseline = 35.8; control = 13.6, *p* = 0.003) [39], and another one found enhancement in the Personal Wellbeing Index (PWI) (mean difference for intervention group from baseline = 11.43; control = 5.47, *p* = 0.0001) [44]. The data was not compatible with the meta-analysis.

*(5) Self-Efficacy*. The data of 119 participants were pooled from 2 studies [44, 48] to reveal a significant benefit of exercise on self-efficacy (SMD = 6.70; CI = 0.90, 12.50; *p* = 0.02). See Figure 6(b).

## 4. Discussion

This systematic review and meta-analysis represents the primary attempt to comprehensively synthesize and analyze the existing evidence on a range of exercise types for older adults with depression and identify which types of exercise could be prescribed to achieve specific health outcomes. This review endorses the recommendations of the National Institute for Health and Clinical Excellence guidelines in support of exercise for this population [15]. It further extends our knowledge on this topic by providing valuable insights into the specific types of exercise that yield the most significant advantages in relation to specific health-related outcomes.

Four distinct categories of exercise have been studied in this population, which include aerobic, tai chi/qigong, multicomponent, and strengthening exercise programs. It seems that each type of exercise program offers distinct health benefits. Evidence indicates that tai chi/qigong exercise offers the most notable advantages in enhancing multiple health outcomes that include physical health and function, mental health and wellbeing, self-efficacy, cognitive function, and quality of life. Multicomponent exercise is of limited benefit to depression or cognitive function, but favorable for physical health and the physical health component of quality-of-life measures. Similarly, aerobic exercise can improve mental health wellbeing, and physical function, but not cognitive function, nor anxiety. Strengthening exercises do not appear to be effective for older adults with depression in any of the reported health outcomes.

However, caution must be applied when drawing definitive conclusions about the specific effects of exercise programs due to the small number of studies available for some types of exercise and the heterogeneity of the interventions and the outcome tools used to measure effectiveness. This may contribute to the inconsistency of our findings in relation to a previous review [49]. Bridle et al. reviewed the effect of exercise on depressed older adults and found that multicomponent exercise can reduce depression but that there was insufficient evidence for tai chi and qigong exercise [49]. However, our study supports the positive effect of tai chi and qigong exercises. The difference is that Bridle et al. include a small number of studies in their systematic review, but we include more studies, making our study stronger with a large number of evidence. Our finding confirms those of Park et al. who report the positive benefit of tai chi and qigong exercises on depression [18].

Previous reviews have focused on limited exercise types [19, 23] or limited outcomes [20–23], which restrict their findings. To overcome this narrow focus, we facilitated comparisons across all forms of exercise and health-related outcomes in depressed older populations. To minimize bias and facilitate between-group comparisons, we specifically included controlled trials; however, the quality of some of these studies was poor; the review was hindered by the incongruity of the available evidence; and meta-analysis was limited due to unusable data. Thus, caution must be taken in interpreting these findings as they are prone to type I and type II errors. In the future, researchers must adopt standardized approaches regarding exercise type, dosage, and outcome measurement to facilitate the comparison of findings across studies. Additionally, it is important to acknowledge that our study sample predominantly consisted of females, which may be representative of the older population and may limit the generalizability to male populations. Moreover, our review has a time constraint as we only looked at studies up to 2020. This is because the delivery of exercise changed to reduce personal contact during and after COVID, and this might mean that we did not include some relevant studies. Lastly, this review may have overlooked relevant studies published in languages other than English due to the restriction of our search criteria.

The lack of comprehensive and methodologically rigorous research on the effects of physical exercise in older adults is a significant limitation that hinders both the clinical application of exercise interventions and the development of evidence-based policies.

## 5. Conclusion

This review demonstrates that exercise in older depressed adults has positive effects on a range of health-related outcomes, which appear to be specific to the type of exercise performed. We encourage clinicians to carefully consider the desired health benefits to ensure that the most appropriate exercise is prescribed.

## Figures and Tables

**Table 1:** Quality assessment of the included studies using the PEDro scale.

Study	Criteria item											
	1	2	3	4	5	6	7	8	9	10	11	Total/10
Aguiñaga et al. (2018) [34]	No	1	0	1	0	0	0	0	0	1	1	4
Brenes et al. (2007) [35]	Yes	1	0	1	0	0	1	1	1	1	1	7
Huang et al. (2015) [36]	No	1	0	1	0	0	1	1	0	1	1	6
Jin et al. (2019) [37]	Yes	0	0	1	0	0	0	0	0	1	1	3
Kerse et al. (2010) [38]	Yes	1	1	1	0	0	1	1	0	1	1	7
Lavretsky et al. (2011) [39]	Yes	1	1	1	0	0	1	1	1	1	1	8
Lok et al. (2017) [43]	Yes	1	0	1	0	0	0	1	0	1	1	5
Makizako et al. (2020) [40]	Yes	1	0	1	0	0	0	1	1	1	1	6
Murri et al. (2018) [46]	Yes	1	0	1	0	0	0	0	0	1	1	4
Sims et al. (2006) [47]	Yes	1	1	1	0	0	0	0	1	1	1	6
Singh et al. (2001) [41]	Yes	1	1	1	0	0	1	1	1	1	1	8
Tanaka et al. (2016) [42]	Yes	0	0	1	0	0	0	1	0	1	1	4
Tsang et al. (2006) [44]	Yes	1	0	1	0	0	1	0	0	1	1	5
Tsang et al. (2013) [48]	Yes	1	0	1	0	0	1	1	1	1	1	7
Underwood et al. (2013) [45]	Yes	1	0	1	0	0	1	0	0	1	1	5
Total	13	13	4	15	0	0	8	9	6	15	15	85

Yes = 1. No = 0. Item 1 eligibility criterion does not contribute to total score. Criteria items: 1, eligibility criteria specified; 2, random allocation; 3, concealed allocation; 4, baseline similarity; 5, participant blinding; 6, therapist blinding; 7, assessor blinding; 8, outcomes measured; 9, the intervention provided; 10, between-group comparisons; and 11, outcomes reported.

**Table 2:** Study characteristics.

Author (year)	Study design	Sample size (*n*) Female percentage (%)	Source of participants Age (mean/range)	Experimental intervention (EG) and dosage	Mode of delivery	Comparison intervention (CG) and dosage	Duration (weeks)	Follow-up	Main outcomes and measurement tool	Other outcomes: tool	Main findings
Aguiñaga et al. (2018) [34]	RCT	307 76.8%	Community 70.5	Multicomponent exercises (flexibility, toning, and balance DVD exercises+telephone support calls) 3×/week progress to 4×/week+biweekly telephone support calls	Home-based (DVD)	DVD on healthy aging+telephone support calls 85 min DVD+biweekly calls	24	None	Depression and anxiety: HADS	Leisure time physical activity: GLTEQ Physical self-worth: PSW	EG had significantly greater reductions in depression and anxiety (*d* = 1.66 and 2.90) than CG (*d* = 0.77 and 0.73). The improvement in depression was partly mediated by increased physical self-worth (*β* = 0.13, *p* = 0.02)

Brenes et al. (2007) [35]	RCT	37 62.1%	Community and residential care 74.6	Multicomponent exercise (aerobic and strength exercises) 60 min 3×/week Medication group: open-label sertraline 25 mg/day progressed to 150 mg/day	Supervised	Usual care+telephone call in weeks 2, 6, 10, and 14	16	None	Depression: HDRS and GDS Quality of life: SF-36 (mental health scale)	Physical function: 23-item questionnaire of physical disability and SF-36 (physical health scale)	A trend for both EG and sertraline to be superior to CG in reducing depression (EG: *p* = 0.09, mean = 0.96; sertraline: *p* = 0.06, mean = 1.56) and mental health component of the SF-36 (*F* [2, 26] = 3.02, *p* = 0.07), with significant improvement in EG compared with CG (*p* = 0.02; ES = 0.95) The trend for an effect on physical disability in EG (*p* = 0.19, mean = 0.22)

Huang et al. (2015) [36]	RCT	57 52.6%	Community 76.6	Multicomponent exercise (range of motion, aerobic, and strength exercise) 50 min, 3×/week Cognitive behavioral therapy (CBT) group 60-80 min, 1×/week for CBT	Supervised group class	Usual care 60–80 min	12	6 months 9 months	Depression: GDS	Physical function: 6MWT Quality of life: SF-36 Social support: ISSB	Pre-posttest, EG had decreased GDS-15 scores at 3 time points (*p* = 0.003, 0.012, and 0.037), greater 6MWT distance (*p* = 0.023), better QoL (*p* < 0.001), and perceived support (*p* < 0.001). The CBT group had significantly less depression (*p* = 0.009) at 6 months and better perceived support (*p* < 0.001)

Jin et al. (2019) [37]	RCT	30 100%	Residential care 79.7	Walking group+resistance exercises+arthritis management education led by a nurse 20 min walking at 40-50% of max heart rate, 2×/week 3×/week resistance exercises 1×/month for 3 months then 2×/month	Supervised group exercise	Sedentary lifestyle+arthritis management education 1×/month for 3 months then 2×/month	24	None	Depression: SGDS-K	Physical function: 6MWT, handgrip strength (kg), 2.44-meter up-and-go (sec), sit and reach test (cm)	Repeated measures analysis of variance showed a significant group by time interaction in favor of the EG for depression (*p* < 0.001), strength (*p* = 0.030), 6MWT distance (*p* = 0.001), TUG time (*p* < 0.001), and handgrip strength (*p* = 0.005)

Kerse et al. (2010) [38]	RCT	193 58.5%	Community 81.1	Walking program+progressive resistance lower limb strengthening and balance training 30 min, 3×/week 60 min, every 2-3 weeks for 3 months then monthly telephone calls for 2 months	Home-based exercises+individual instruction by a trained nurse	Active social contact visits only 60 min, every 2-3 weeks for 3 months then monthly phone calls for 2 months	24	12 months	Physical function: SPPB and NEADL	Quality of life: SF-36 Depression: GDS Physical activity: AHSPAQ	There were no between-group differences. Mood and mental health-related quality of life improved for both groups

Lavretsky et al. (2011) [39]	RCT	73 61.6%	Community 70.6	Tai chi+medication (escitalopram) 120 min, 1×/week+escitalopram 10-20 mg/day	Supervised group exercise	Health education+escitalopram 120 min 1×/week+escitalopram 10-20 mg/day	10	None	Depression: HDRS	Cognitive MMSE+cognitive battery Medical comorbidity: risk factor prediction chart Global chronic illness burden: CIRS-G Anxiety: HAS Quality of life: SF-36	Improvement in depression was greater in EG compared to CG (*p* < 0.05). Significant improvement in EG in SF-36 (*p* = 0.02) and cognition (*p* < 0.05) compared to CG

Lok et al. (2017) [43]	RCT	80 45%	Residential care NR, but states >65 yrs	Physical activity (rhythmic exercises)+walking 40 min physical activity and 30 min free walking, 4×/week	Group class	Usual care	10	None	Depression: BDI Quality of life: SF-36	None	Between-group significant difference in the BDI favoring the EG (*p* = 0.005). 8 subscales of the SF-36 significantly improved only in the EG (*p* < 0.05)

Makizako et al. (2020) [40]	RCT	89 50.5%	Community 73	Aerobic, strength, balance, and dual-task training 90 min, weekly Horticultural group: cultivating, growing, and harvesting 60-90 min weekly	Group class	Education only 90 min 2×/week	2	12 months	Depression: GDS Memory performance: WMS-R+wordlist memory tasks	Physical function: 2MWT Cognitive function: VFT+TMT Alzheimer's disease VSRAD Quality of life: HRQOL	No significant between-group differences in depression postintervention (*p* = 0.744) or follow-up (*p* = 0.741). Significant difference favoring EG for 2MWT (*p* = 0.016) Significant differences in memory: immediate (*p* < 0.001) and delayed (*p* = 0.007) postintervention favoring EG. At follow-up, significant between-group differences in immediate recall (*p* = 0.014) remained, but delayed recall did not (*p* = 0.315)

Murri et al. (2018) [46]	RCT	121 71%	Mental health hospital 75.3	Medication+nonprogressive exercise (strength, balance, respiration, and motor coordination, and comprised both mat work and instrumental exercises) OR medication+progressive aerobic exercise 60 min, 3×/week+50 mg (or more) sertraline	Supervised	Antidepressants only (no exercise) NR	24	None	Depression: HAM-D	—	Lower unadjusted scores for affective and vegetative symptoms (both *p* < 0.025) for EG compared to CG

Sims et al. (2006) [47]	RCT	30 70%	Medical practices 74.7	Strength training+weekly telephone call 3×/week	Supervised	Advice only NR	10	6 months	Depression: GDS PGCMS Quality of life: the WHOQoL-BREF questionnaire Physical activity: PASE Functional health status: HAP		At six months, there was a trend for the EG to have lower GDS scores than the CG, but this finding did not reach significance (*p* = 0.08). More of the EG (57%) had a reduction in depression compared to 44% of the CG

Singh et al. (2001) [41]	RCT	32 63%	Community 71.3	Resistance training 3×/week (phase one and two) Weekly telephone calls (phase two only)	Phase one (weeks 1-10): supervised in laboratory. Phase two (weeks 11-20): unsupervised at the laboratory, health setting, or home+written log and telephone calls Phase three (months 6-26): no contact or study requirements	Phase one: health education lectures Phase two: telephone calls by the investigator Phase three: no contact or study requirements 60 min, 2×/week (phase one) Weekly telephone calls (phase two)	20	26 months	Depression: BDI	Self-efficacy and morale: PGCMS	The BDI was significantly reduced at both 20 weeks and 26 months in EG compared with CG (*p* < 0.05)

Tanaka et al. (2016) [42]	RCT	40 100%	Community 78.3	Japanese drum exercise 40 min 1×/week	Supervised	Usual care	12	None	Mood: POMS-SF and PGCMS Depression: GDS Body composition: bioelectrical impedance data acquisition system Physical function: TUG, SSTw, and handgrip strength (kg) Cognitive function: TMT and MMSE	—	No significant between-group differences in any of the psychophysiological or physical fitness measures after the intervention

Tsang et al. (2006) [44]	RCT	82 80.5%	Residential care 82.4	Qigong exercise 30-45 min, 3×/week	NR	Newspaper reading 30-45 min, 3×/week	16	4 weeks 8 weeks	Depression: GDS, the Chinese version Self-efficacy: CGSS QOL: PWI Psychological wellbeing: self-concept scale: ASSEI Perceived Benefit Questionnaire	—	Between-group differences in favor of the EG for depression (*F* = 69.98, *p* = 0.001), self-efficacy (*F* = 70.41, *p* = 0.001), QoL (*F* = 54.06, *p* = 0.001), and perception of general health (*F* = 8.14, *p* = 0.006)

Tsang et al. (2013) [48]	RCT	38 68.4%	Geriatric day clinics and residential care 80.2	Qigong exercise 45 min 3×/week	Supervised	Newspaper reading+discussion group. 45 min 3×/week	12	4 weeks 8 weeks	Depression: GDS and HDRS Self-efficacy: CGSS Psychological wellbeing: self-concept: SQCPD Physical function: handgrip strength (kg)		Significant reduction in depression in EG (*F* = 11.68, *p* < 0.025). Improvement in self-efficacy (*F* = 4.30, *p* < 0.050), self-concept of physical wellbeing (*F* = 6.82, *p* < 0.025), and right-hand grip strength (*F* = 5.25, *p* = 0.034) compared to CG

Underwood et al. (2013) [45]	RCT	891 76%	Residential care 86.5	Strength and aerobic training+depression awareness training 45 min, 2×/week	Supervised	Depression awareness training 1×/week	12 months		Depression: GDS	Cognitive function: MMSE QOL: EQ-5D Physical function: SPPB	No statistically significant, or clinically important, between-group differences in depression, at 12 months, (adjusted mean difference 0.13 points, 95% CI –0·33 to 0·60, *p* = 0.5758). No between-group difference in any secondary outcomes

EG: exercise group; CG: control group; cm: centimeters; kg: kilos; NR: not reported; RCT: randomized control trial; sec: seconds; 2MWT: 2-minute walking test; 6MWT: 6-minute walk test; AHSPAQ: Auckland Heart Study Physical Activity Questionnaire; ASSEI: self-concept scale; BDI: Beck Depression Inventory; CGSS: Chinese General Self-Efficacy Scale; CIRS-G: Cumulative Illness Rating Scale-Geriatric; EQ-5D: European quality of life-5 dimension instrument; GDS: Geriatric Depression Scale; GLTEQ: Godin Leisure Time Exercise Questionnaire; HAP: Human Activity Profile; HAM-D: Hamilton Depression Rating Scale; HADS: Hospital Anxiety and Depression Scale; HAS: Hamilton Anxiety Scale; HDRS: Hamilton Depression Rating Scale; HRQOL: Short Form Health Survey-12 (SF-12); ISSB: Inventory of Socially Supportive Behavior; MMSE: Mini-Mental Scale Examination; NEADL: self-report Nottingham Extended Activities of Daily Living; PASE: Physical Activity Scale for the Elderly; PGCMS: Philadelphia Geriatric Center Morale Scale; POMS-SF: Short form of the Profile of Mood States; PSW: physical self-worth; PWI: Personal Well-Being Index; SF-36: 36-Item Short Form Survey; SSTw: shuttle stamina test walking; SPPB: Short Physical Performance Battery; SGDS-K: The Korean version of the short form of the Geriatric Depression Scale; SQCPD: Self-concept Questionnaire for Hong Kong Chinese Adults with Physical Disabilities; TUG: timed up and go; TMT: trail making test; VFT: verbal fluency test; VSRAD: voxel-based specific regional analysis system for Alzheimer's disease; WMS-R: Wechsler Memory Scale-Revised; WHOQoL: World Health Organization Quality of Life.

**Table 3:** Summary of results: number of studies in each category with references.

Exercise type	Outcome measure category	Numbers of studies	Significant improvement (vs. control group) at postintervention measurement (*n*)	Studies that reported follow-up^a^	Significant improvements reported at follow-up^b^
Mixed exercise	Physical health and function	*n* = 7 [34–38, 40, 45]	*n* = 5 [34–37, 40]	*n* = 2 [36, 40]	N/A
	Cognitive function	*n* = 2 [40, 45]	*n* = 1 [40]	*n* = 1 [40]	*n* = 1 [40]
	Mental health and wellbeing	*n* = 8 [34–38, 40, 43, 45]	*n* = 4 [35–37, 43]	*n* = 1 [36]	*n* = 1 [36]
	Quality of life	*n* = 6 [35–36, 38, 40, 43, 45]	*n* = 3 [43, 35, 36]	*n* = 1 [36]	N/A

Aerobic exercise	Physical health and function	*n* = 1 [42]	*n* = 1 [42]	N/A	N/A
	Cognitive function	*n* = 1 [42]	N/A	N/A	N/A
	Mental health and wellbeing	*n* = 2 [42, 46]⁣^∗^	*n* = 1 [46]⁣^∗^	N/A	N/A

Strength exercise	Physical health and function	*n* = 1 [47]	N/A	N/A	N/A
	Mental health and wellbeing	*n* = 2 [41, 47]	*n* = 2 [41, 47]	*n* = 2 [41, 47]	*n* = 2 [41, 47]
	Quality of life	*n* = 1 [47]	N/A	N/A	N/A

Tai chi exercise/qigong exercise	Physical health and function	*n* = 1 [48]	*n* = 1 [48]	*n* = 1 [48]	N/A
	Cognitive function	*n* = 1 [39]	N/A	N/A	N/A
	Mental health and wellbeing	*n* = 3 [39, 44, 48]	*n* = 3 [39, 44, 48]	*n* = 2 [44, 48]	*n* = 2 [44, 48]
	Quality of life	*n* = 2 [39, 44]	*n* = 2 [39, 44]	*n* = 1 [44]	*n* = 1 [44]

Some studies used more than one tool to measure the same outcome. Not all the tools necessarily showed improvement. ^a^Follow-up data collection varied from 1 month to 24 months. ^b^Significant improvement was reported at the first follow-up for each study. ⁣^∗^This author has two exercise interventions, multicomponent and aerobic exercise, but has combined the results of both cohorts into one group for reporting.

## Data Availability

All data used in this systematic review had been published in original articles from which it was extracted. In some cases, conversions and calculations were conducted to create homogeneity of the data. All data use in meta-analysis will be stored for years in accordance with ethics guidelines and is available on request.

## Supplementary Materials

Supplementary Materials Example search engine terms as used in MEDLINE.
