# Supplementary material for: The Effects of Various Types of Physical Exercise on Health Outcomes in Older Adults with Depression: A Systematic Review and Meta-Analysis of Controlled Trials
Source: Depress Anxiety. 2024 Jun 19;2024:9363464. doi: 10.1155/2024/9363464 (PMC11918806; doi:10.1155/2024/9363464)
Supplement: Supplementary Materials — Example search engine terms as used in MEDLINE. [file 9363464.f1.pdf]

Appendix 1: Example Search: Ovid Medline

#	Search Terms	Results
1	exp Neurocognitive Disorders/	248632
2	cognitive impairment.mp.	56615
3	Dementia/	49945
4	Alzheimer Disease/	91753
5	exp Parkinsonian Disorders/	78740
6	exp Multiple Sclerosis/	57760
7	exp Stroke/ or Healthy aged.mp. [mp=title, abstract, original title, name of substance word, subject heading word, floating sub-heading word, keyword heading word, organism supplementary concept word, protocol supplementary concept word, rare disease supplementary concept word, unique identifier, synonyms]	131913
8	musculoskeletal diseases/ or exp arthralgia/ or arthritis/ or exp osteoarthritis/	118244
9	anxiety disorders/ or mood disorders/ or depressive disorder/ or healthy aged.mp. [mp=title, abstract, original title, name of substance word, subject heading word, floating sub-heading word, keyword heading word, organism supplementary concept word, protocol supplementary concept word, rare disease supplementary concept word, unique identifier, synonyms]	106985
10	1 or 2 or 3 or 4 or 5 or 6 or 7 or 8 or 9	731094
11	Exercise/ or Exercise Therapy/ or Exercise recommendation*/ or exercise.mp.	344290
12	physical activit*.mp.	110488
13	11 or 12	402618
14	exp Aged/	3068314
15	((older or elderly or senior) adj5 (adult* or men or women or male* or female* or man or woman or person* or people or population or citizen*)).mp.	257174
16	geriatric*.mp.	101400
17	older age*.mp.	46014
18	14 or 15 or 16 or 17	3203380
19	10 and 13 and 18	8356
20	limit 19 to english language	7912
21	limit 20 to yr="1990 -Current"	7821
